# Towards precision medicine: advances in 5-hydroxymethylcytosine cancer biomarker discovery in liquid biopsy

**DOI:** 10.1186/s40880-019-0356-x

**Published:** 2019-03-29

**Authors:** Chang Zeng, Emily Kunce Stroup, Zhou Zhang, Brian C.-H. Chiu, Wei Zhang

**Affiliations:** 1 Driskill Graduate Program in Life Sciences, Northwestern University Feinberg School of Medicine, Chicago, IL 60611 USA; 2 Department of Preventive Medicine, Northwestern University Feinberg School of Medicine, 680 N. Lake Shore Dr., Suite 1400, Chicago, IL 60611 USA; 3 Department of Public Health Sciences, University of Chicago, Chicago, IL 60637 USA; 4 The Robert H. Lurie Comprehensive Cancer Center, Northwestern University Feinberg School of Medicine, Chicago, IL 60611 USA; 5 Institute of Precision Medicine, Jining Medical University, Jining, 272067 Shandong P. R. China

**Keywords:** Liquid biopsy, Cell-free DNA, Epigenetics, Cancer biomarker, 5-Hydroxymethylcytosine

## Abstract

Robust and clinically convenient biomarkers for cancer diagnosis, early detection, and prognosis have great potential to improve patient survival and are the key to precision medicine. The advent of next-generation sequencing technologies enables a more sensitive and comprehensive profiling of genetic and epigenetic information in tumor-derived materials. Researchers are now able to monitor the dynamics of tumorigenesis in new dimensions, such as using circulating cell-free DNA (cfDNA) and tumor DNA (ctDNA). Mutation-based assays in liquid biopsy cannot always provide consistent results across studies due partly to intra- and inter-tumoral heterogeneity as well as technical limitations. In contrast, epigenetic analysis of patient-derived cfDNA is a promising alternative, especially for early detection and disease surveillance, because epigenetic modifications are tissue-specific and reflect the dynamic process of cancer progression. Therefore, cfDNA-based epigenetic assays are emerging to be a highly sensitive, minimally invasive tool for cancer diagnosis and prognosis with great potential in future precise care of cancer patients. The major obstacle for applying epigenetic analysis of cfDNA, however, has been the lack of enabling techniques with high sensitivity and technical robustness. In this review, we summarized the advances in epigenome-wide profiling of 5-hydroxymethylcytosine (5hmC) in cfDNA, focusing on the detection approaches and potential role as biomarkers in different cancer types.

## Background

Robust and clinically convenient cancer biomarkers are of great importance for the successful delivery of precision medicine and better clinical care for cancer patients. Firstly, the latency for cancers is long, and cancer patients usually exhibit symptoms at advanced stages when curative treatment may no longer be available. Therefore, screening and early detection of cancers at asymptomatic and/or curable stages are especially critical in improving patients’ survival and quality of life [1–3], as well as reducing the burden of healthcare system. Secondly, the dynamics of tumorigenesis is the main hurdle to effective treatments in cancer patients [4] and remains a grand challenge for cancer precision medicine. Tumor heterogeneity and clonal evolution are the two major consequences of the dynamics of tumorigenesis, which can simultaneously drive tumor evolution [5], posing challenges in the selection of anticancer drugs as well as the optimal doses of these drugs. Thirdly, patients with metastatic cancers of unknown primary sites usually have poor prognosis and dismal survival rate because site-specific targeted therapies are not effective [6, 7]. To reduce cancer mortality and improve the overall quality of healthcare outcomes and well-being of the patients, there is an urgent need to develop minimally-invasive biomarkers that are sensitive and specific enough for clinical applications such as early cancer detection, longitudinal surveillance of dynamic tumor progression under drug treatment, and the selection of targeted therapies for cancers of unknown primary sites. In this review, we overview the current states of molecular biomarkers in cancer diagnosis and prognosis, as well as discuss the potential benefits and limitations of the new approaches for detecting a novel class of epigenetic biomarkers, 5-hydroxymethylcytosine (5hmC) biomarkers, in cell-free DNA (cfDNA). Furthermore, we provide an update and current perspectives for 5hmC alterations in patients with different cancer types and discuss their potential role as cancer biomarkers. Finally, we point out the improvements and future work required to make these biomarkers effective in the clinic.

## Current strategies of cancer biomarker discovery

### Tissue biopsy versus liquid biopsy

Tumor tissues are the gold-standard sources for identifying cancer-specific biomarkers. However, tissue biopsy has some intrinsic limitations as it can be invasive and clinically risky. Tissue biopsy usually requires surgical resection to obtain tumor tissues, and surgery entails risks such as bleeding and infection [8, 9]. In addition, information acquired from a single-region tissue biopsy only provides a spatially limited snap-shot of a tumor and might fail to reflect the intra-tumor heterogeneity [4, 10]. This could lead to an inaccurate diagnosis of cancer type and stage or unreliable prognostic assessment of relapse risk and survival [11]. Multi-region tissue biopsy offers an alternative way to capture the intra-tumor heterogeneity [10, 12]; however, its clinical application is limited due to the volume and accessibility of tumor tissues [9]. Furthermore, drug treatment confers selective pressure on tumor cells, resulting in adaptive clonal evolution and possibly subsequent drug resistance [4, 13, 14]. While longitudinal profiling of tumor heterogeneity provides valuable information in treatment response evaluation and therapy optimization, this again is of limited clinical feasibility given the difficulty of obtaining tumor tissues for multiple time points [15].

**Fig. 1:**
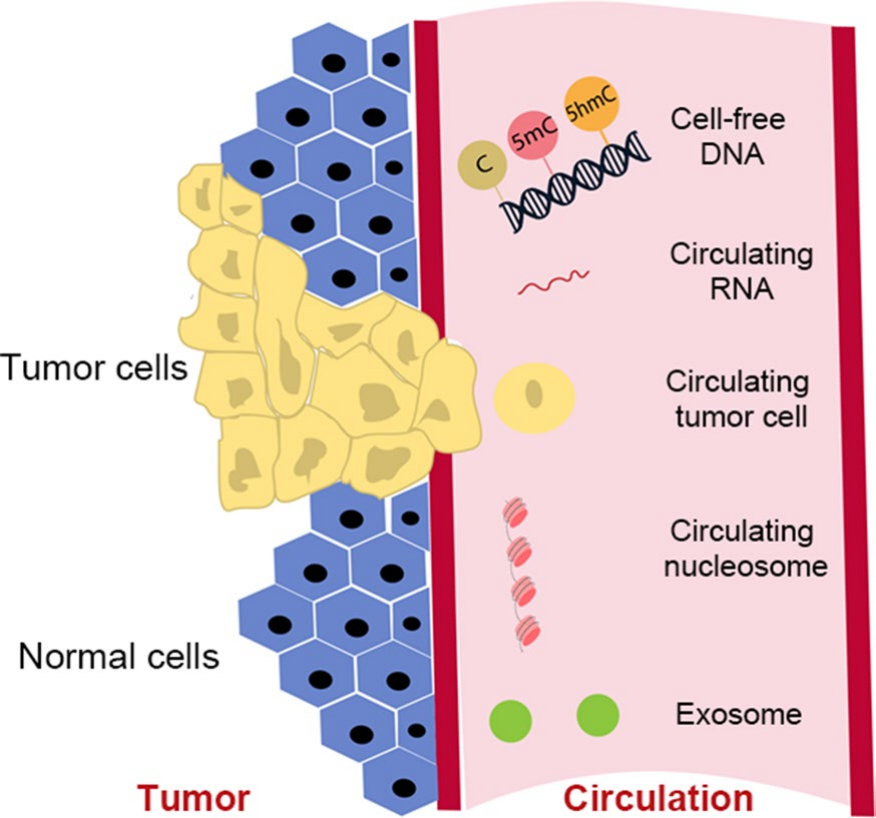
The origins of biomarkers for liquid biopsy. Molecular alterations of circulating materials, such as cell-free DNA, circulating tumor cells, exosomes, and circulating nucleosomes and RNA, can be used as biomarkers for cancer diagnosis and prognosis. Genetic biomarkers can be identified via mutational profiling of nucleic acids extracted from circulating tumor cells. Epigenetic biomarkers can be obtained via methylation profiling and nucleosome foot printing of cell-free DNA, circulating tumor cells, and circulating nucleosomes and RNA

In contrast, liquid biopsy is emerging as a minimally invasive tool with great potential in cancer management. It uses circulating materials such as cfDNA, circulating tumor cells (CTCs), and exosomes to detect molecular alterations that are indicative of cancer progression. cfDNA is fragmented cellular DNA released into the bloodstream by cells undergoing apoptosis and necrosis, and possibly through active secretion [16, 17]. In healthy individuals, cfDNA is primarily derived from apoptotic hematopoietic cells [18], whereas, in cancer patients, cfDNA can be of tumor tissue and tumor microenvironment origin, reflecting the genetic and epigenetic alterations of tumor tissues and their corresponding microenvironment [19]. As a result, cfDNA has been extensively studied recently, especially in the field of early cancer detection, cancer staging and subtyping, and disease surveillance. The origins of biomarkers for liquid biopsy are summarized in Fig. 1.

### Genetic biomarkers versus epigenetic biomarkers

Clinical trials based on mutational profiling of circulating tumor DNA (ctDNA; cfDNA of tumor origin) are increasing. Several studies have demonstrated the feasibility of applying ctDNA analysis to capture the heterogeneity of cancer evolution, therefore, making ctDNA analysis a tool for cancer surveillance and care [20]. For example, longitudinal sampling of ctDNA levels not only indicated the presence of cancer but also had good association with therapy response and disease progression in pancreatic cancer [21]. Additionally, driver mutations identified in ctDNA samples showed a high concordance with that from matched tumor samples in prostate cancer [19] and multiple myeloma [22]. In metastatic breast cancer, estrogen receptor 1 (*ESR1*) mutations are responsible for the resistance to aromatase inhibitors [23]. The mutational profile of *ESR1* in ctDNA was associated with that in tumor tissues and therefore reflected the clonal evolution of breast cancer under the treatment of aromatase inhibitors [23]. This evidence indicates that *ESR1* mutations in ctDNA are potential biomarkers in treatment monitoring. Despite several studies have demonstrated that the mutational signatures in ctDNA were consistent with those in corresponding tumor tissues, there is currently insufficient evidence of clinical validity and utility for the majority of ctDNA-based mutational assays in advanced cancer, and there is no evidence supports that they can be applied to early cancer detection [20, 24, 25].

In addition, merely relying on the identification of tumor-derived driver mutations in ctDNA cannot capture the whole complexity of tumor biology [26]. Unlike mutations, the reversible epigenetic modifications are more plastic and can reflect the changes of tumor microenvironment and tissue of origin [27, 28]. Epigenetic modifications such as DNA methylation may represent a novel and promising analytical tool for biomarker discovery with broad potential applications in risk assessment, early cancer detection, prognosis, and prediction of response to therapy [29–31]. To date, DNA methylation-based assay, Epi proColon, has been approved by the US Food and Drug Administration (USFDA) for colon cancer detection [32]. In the early stages of carcinogenesis, many epigenetic changes have occurred in normal tissues before somatic mutations and histopathological changes can be detected [33]. Therefore, epigenetic analysis of cfDNA combined with mutation-based analysis may contribute to a better understanding of the interplay across molecular alterations in the cancer genome, epigenome, and tumor microenvironment in tumor heterogeneity and clonal evolution [27, 28, 30, 34–36].

Despite the promises, the applications of these genetic or epigenetic biomarkers in population screening and very early stage cancer detection can still be challenging. Like traditional biomarkers, they also suffer from the same issues of low sensitivity and specificity because of the limited amount of circulating materials and the noise in the detection [37].

## Advances in epigenetic cancer biomarker discovery in liquid biopsy

The most extensively studied epigenetic feature for cancer biomarker discovery in cfDNA is DNA methylation, especially the 5-methylcytosine (5mC) modification at CpG dinucleotides [29, 35, 38–41]. In hepatocellular carcinoma, 5mC biomarkers derived from ctDNA showed better diagnostic and prognostic values than currently used indicators (such as serum-based alpha-fetoprotein [AFP] and TNM staging) [35]. In addition, repetitive elements such as long intersperse nucleotide element 1 (LINE-1) and Alu are known proxies for global DNA methylation [42]. In diffuse large B cell lymphoma, LINE-1 methylation in cfDNA has been shown to be strongly associated with clinical outcomes, demonstrating its potential as a prognostic biomarker [43]. Another approach in 5mC biomarker discovery is to identify tissue-specific methylation haplotypes as biomarkers to estimate tumor burden and tissue-of-origin in cfDNA [40]. These multi-CpG haplotypes have been shown to outperform the traditional single-CpG methylation biomarker in cancer classification [40]. Recently, other epigenetic features such as 5-hydroxymethylcytosine (5hmC) and nucleosome positioning and occupancy on cfDNA have also been utilized to infer tissue of origin and cancer progression [27, 44–47]. Although genome-wide nucleosome distribution of cfDNA provides valuable information in the deconvolution of pooled cfDNA to infer tissue of origin, its clinical application has not been extensively studied [27, 48]. In this review, we summarize the advances in the genome-wide profiling of 5hmC dynamics in cfDNA for cancer biomarker discovery based on the unique features and distinct biological functions of modified cytosines.

### 5-Hydroxymethylation

In the human genome, 5mC is the most abundant and well-known DNA methylation variant that plays an important role in the regulation of gene expression [49]. The 5mC-associated methylation patterns are usually tumor- and tissue-specific, reflecting the origin of the metastatic tumors and their altered epigenomes [36]. With the mediation of ten-eleven translocation (Tet) proteins, 5mC can be further oxidized to 5hmC, 5-formylcytosine (5fC), and 5-carboxylcytosine (5caC) [50]. Among them, 5hmC is the most abundant and stably oxidized product [51]. In contrast to about 8% of cytosine that is methylated in the human genome, only 0.5%–1% of cytosines are hydroxymethylated. The scarcity of 5hmC in the genome may pose challenges in distinguishing true signals from noise upon sequencing. However, it also has the potential to improve the statistical power in biomarker discovery because of the reduced multiple hypothesis burden [39, 52–54]. Unlike the uniform distribution of 5mC outside of the promoter regions, satellites, and repeat DNA sequences [55], 5hmC has distinct distributions across different functional regions, and its abundance varies across different tissues and cell types [56, 57], with tissue type playing a dominant role in determining the distribution patterns of 5hmC [58]. 5hmC is enriched primarily in the distal regulatory regions, gene bodies of actively expressing genes and promoters, indicating its connection with active transcription [59]. Genome-wide analysis of 5mC has indicated the global hypomethylation pattern in tumor tissues, whereas depletion of 5hmC has also been associated with the hypermethylation of gene bodies in various cancers [58, 60, 61]. Significant enrichment of 5hmC is observed in both tissue-specific and cancer-specific differentially methylated regions as compared with that of 5mC [62]. Thus, genome-wide analysis of 5hmC dynamics can further refine our understanding of the relationship between cancer and methylome.

### Enabling technologies for profiling 5hmc

Because 5hmC and 5mC dynamics can be informative of tumorigenesis, epigenome-wide analysis of cfDNA has also been conducted to identify minimally-invasive cfDNA-derived biomarkers for better cancer management [35, 45, 46, 63–66]. An overview of the most common genome-wide 5hmC quantification methods are summarized in Table 1.

**Table 1:** Overview of genome-wide 5hmC quantification methods

Technique	Advantages	Disadvantages
Whole-genome TAB-Seq [70] *(direct measurement of 5hmC)*	Whole-genome Single nucleotide resolution Absolute methylation value	High sequencing depth required Input > 100 ng DNA
Whole-genome oxBS-Seq [71, 74] *(inference of 5hmC)*		
RRBS TAB-Seq [91] *(direct measurement of 5hmC)*	Single nucleotide resolution Absolute methylation value	Targets CpG islands Input > 500 ng DNA
RRBS oxBS-Seq [52] *(inference of 5hmC)*	~ 60 million PE reads/sample	Input > 500 ng DNA
Nano-hmC-Seal [53] *(direct measurement of 5hmC)*	Input 5 ng DNA Excellent genomic coverage High specificity for 5hmC ~ 30 million PE reads/sample	Lower resolution

5hmC, 5-hydroxymethlcytosine; TAB-Seq, Tet-assisted bisulfite sequencing; oxBS-Seq, oxidative bisulfite sequencing; PE, paired-end

Bisulfite-based whole-genome sequencing and reduced representation bisulfite sequencing (RRBS) are conventional methods for methylation profiling and have been applied in biomarker discovery with cfDNA [40, 67]. However, there are several limitations of applying bisulfite-based methods on cfDNA. First, traditional bisulfite-based methods cannot distinguish between 5mC and 5hmC, and thus are not capable of capturing the dynamics of these two distinct modification types [68, 69]. Second, although modified bisulfite-based methods such as Tet-assisted bisulfite sequencing (TAB-seq) [70] and oxidative bisulfite sequencing (oxBS-seq) [71] can detect and quantify 5hmC at nucleotide resolution, they require an amount of DNA input (> 100 ng) that is not feasible for cfDNA from blood samples (1–2 ng cfDNA from 2 to 3 mL of plasma). This makes its application in early cancer detection challenging [72]. Third, bisulfite-based methods require a high sequencing depth, which is costly and further limits their application in the clinical setting. Restriction enzyme-based methods like reduced representation 5-hydroxymethylcytosine profiling rely on the efficiency of enzymes. As a result, their detection capacity can be limited as inefficient digestion might result in the loss of information on certain 5hmC sites [73]. In contrast, enrichment-based methods such as hME-Seal [53] and the nano-hmC-Seal [59] rely on the selective chemical binding of 5hmC to enrich for 5hmC-containing DNA fragments. These fragments are then sequenced to obtain genome-wide information of 5hmC [53, 59]. Because the size of these chemically selected fragments is a major determinant of the resolution of 5hmC mapping [56], these enrichment-based methods can provide good coverage and high specificity despite low resolution [74]. Given that these enrichment-based approaches can achieve a balance between cost and detection capacity, they are likely to have great potential to be applied widely in the cfDNA-based liquid biopsy in cancer management for large cohorts in the clinical setting [75]. However, lack of a consensus computational framework to analyze these enrichment-based sequencing data may also hamper the interpretation of the results from the clinicians’ end. Although 5hmC-Seal technology is the state-of-the-art in genome-wide profiling of 5hmC dynamics in cfDNA, it relies on relative abundance to infer absolute modification levels and cannot provide single-base resolution 5hmC information. Development of statistical approaches to infer base resolution 5hmC modification levels from enrichment counts would provide more insights into the dynamics of 5hmC. In addition, many of the intrinsic features of 5hmC enrichment-based sequencing data must be taken into consideration to build this computational framework. GC content, copy number variation, strand-specific and asymmetric 5hmC distribution need to be statistically corrected before downstream analysis. Therefore, an integrative computational framework must be established for 5hmC-enrichment sequencing data to increase the sensitivity and specificity of 5hmC-derived biomarkers in cancer research and enable their easy clinical application in the future. The computational analysis of 5hmC enrichment-based sequencing data can be decomposed into the following components: (1) modeling (to model 5hmC enrichment signal taking into account factors such as enrichment bias, local density bias, and copy number variations); (2) quantification (to infer modification level from normalized regional count-based enrichment data [76]); (3) construction of cancer prediction models (to identify differentially modified sites or regions among different conditions [76]). Another approach is to construct a model to predict cancer status or stage based on the estimated proportions and the tissue-of-origin of tumor-derived cfDNA in the blood sample [39].

### Advances in 5hmc-based cancer biomarker discovery

Several recent studies have demonstrated that 5hmC signatures in cfDNA are reliable and sensitive epigenetic markers that are indicative of types and stages of cancers [44–46]. These cfDNA-derived 5hmC biomarkers are found to achieve higher detection sensitivity than classical biomarkers [45].

#### Colorectal cancer (CRC)

CRC is a commonly diagnosed cancer [1, 77]. Despite recent declines in CRC incidence in the United States and other developed countries, the incidence and mortality continue to increase in the rest of the world [1]. This reduction in CRC incidence is largely attributed to screening via colonoscopy and to cancer prevention efforts [1, 77]. However, the invasive nature of colonoscopy leads to poor patient compliance. Stool and blood DNA methylation assays based on candidate genes show great diagnostic and prognostic values, but the sensitivities and specificities of the assays vary and are usually inconsistent [78]. In a Chinese cohort study evaluating cfDNA-derived 5hmC analysis in 80 colorectal cancer patients and 90 healthy individuals [45], a total of 989 differentially methylated 5hmC loci in gene bodies were selected as biomarkers to train the machine learning algorithm for cancer classification. The classifier achieved 83% sensitivity and 94% specificity (area under curve [AUC] = 0.95) in the validating dataset (24 patients and 35 controls) and 88% sensitivity and 89% specificity (AUC = 0.94) in another independent validating dataset (32 patients and 37 controls) for cancer classification [45]. The discriminatory performance of these cfDNA-derived 5hmC biomarkers was not only comparable to that of 5hmC tissue biomarkers but also significantly outperformed the current USFDA approved blood-based methylation test Epi proColon [45, 79]. Epi proColon relies on the methylation status of the single gene septin 9 (*SEPT9*) to infer the presence of cancer and can only achieve a detection sensitivity of 0.48 [79]. To be noted, the sensitivity and specificity of the cancer classifier often vary with cancer stages. Because the majority of colorectal cancer patients in this study are at TNM stages III and IV, the performance of this classifier cannot be over-interpreted [45].

#### Gastric cancer (GC)

GC is a common digestive cancer with 26,240 new cases and 10,800 deaths estimated in 2018 in the United States [1]. Early-stage GC is asymptomatic and exhibits high genomic heterogeneity, making endoscopic or surgical biopsy-based molecular testing rather inaccurate and non-representative [80]. Recent studies suggested the potential role of cfDNA-based molecular profiling in future clinical applications such as diagnosis and targeted therapy selection. However, the epigenetic alterations on cfDNA remain understudied [81–83]. A pilot study has explored the 5hmC alterations in cfDNA from GC patients as compared to that from healthy individuals in a Chinese cohort [45]. Patients with GC and controls were divided into discovery (7 patients and 18 controls) and validation groups (25 patients and 35 controls) [45]. In total, 1431 differentially methylated 5hmC loci in gene bodies were identified and trained on the cancer classifier, and the classifier achieved 92% sensitivity and 91% specificity (AUC = 0.93) in the validating dataset and 90% sensitivity and 97% specificity (AUC = 0.97) in another independent validating dataset [45]. Again, consistent with the study in CRC, these cfDNA-derived 5hmC biomarkers performed better than classical early diagnosis biomarkers, such as carcinoembryonic antigen (CEA) and cancer antigen 19-9 (CA19-9), and other epidemiological factors, such as smoking and alcohol [45]. In both CRC and GC, the classifiers derived from cfDNA are disease-, clinical stage-, and cancer type-specific, suggesting their potential values as diagnostic cancer biomarkers [45].

#### Esophageal cancer (EC)

EC is among the five leading causes of cancer-related death in male patients between age 40 and 59 [1]. Similar to GC, evidence for the effects of molecular biomarkers on diagnosis and treatment guidance are limited because of the challenges in detecting genomic alterations or clinical symptoms at early stages and in resolving the discrepancy of genomic profiling between primary tumors and metastatic lesions [81]. One study conducted genome-wide 5hmC profiling in 150 newly diagnosed EC patients and 177 healthy controls in China [46]. The classifier achieved 93.75% sensitivity and 85.71% specificity with an average AUC of 0.947. Consistent with the observations in CRC and GC, the 5hmC signatures on cfDNA were indicative of clinical stages of EC. The probability of predicting cancer based on the 5hmC classifier increased with the progression of cancer stage. In addition, EC patients with lymph node metastases were predicted to have significantly higher cancer probability as compared with patients without lymph node metastases [46].

#### Lung cancer (LC)

LC is the leading cause of cancer-related death in both genders with 83,550 estimated deaths in males and 70,500 estimated deaths in females in 2018 [1]. Unlike the steady increase of 5-year survival rate in most cancer types, the 5-year survival rate of LC patients is the second lowest with little improvement over the past years [1]. Minimally-invasive early LC detection is urgently needed to improve the survival rate because the low-dose computed tomography screening not only fails to provide low false positive results with high predictive values but also requires additional invasive testing procedures afterwards [66, 84]. One study evaluated the diagnostic value of 5hmC signatures in 15 LC patients from China and 40 healthy controls from the United States and found that global cell-free 5hmC levels were gradually depleted during the development from early non-metastatic to late metastatic stages [44]. Another independent study on a Chinese cohort identified 2459 genes with differential 5hmC levels by comparing the genome-wide 5hmC profiles among 66 non-small cell lung cancer patients and 67 healthy individuals [85]. Candidate 5hmC biomarker panel derived from this study was observed to achieve better detection sensitivity as compared with that of known clinical biomarkers such as CEA, carbohydrate antigen 125 (CA125) and neuron-specific enolase (NSE).

#### Multiple myeloma (MM)

MM is the second most common hematological malignancy with profound disruption of epigenomes. A recent proof-of-concept study applied the nano-hmC-Seal technology to explore the potential of cfDNA-derived 5hmC biomarkers to improve the current invasive and expensive bone marrow biopsy-based biomarkers [86]. This study profiled the genome-wide 5hmC modifications in 9 MM patients, 5 patients with premalignant precursor condition defined as monoclonal gammopathy of undetermined significance, 5 patients with another premalignant precursor condition defined as smoldering multiple myeloma, and 19 newly diagnosed, treatment-naïve European American patients as controls. In total, 183 genes containing differential 5hmC loci were identified, and MM patients were separated from the patients in precursor conditions utilizing these 5hmC signatures. In addition, MM patients with different relapse statuses could also be separated using 5hmC signatures [86]. These preliminary findings again highlighted the values of 5hmC signatures as independent diagnostic and prognostic biomarkers in MM.

## Conclusions and future directions

With the biological understanding that 5hmC is at the nexus of glucose metabolism and cancer epigenetics [61], recent studies have demonstrated the clinical prospects of using genome-wide 5hmC dynamics on cfDNA to improve cancer management. However, several challenges remain, limiting the translational success of cfDNA-derived 5hmC biomarkers in clinical settings. Current studies of 5hmC biomarker discovery have been mostly focused on genic regions, partly because of their genomic enrichment pattern and putative gene regulatory role. Expanding 5hmC biomarker discovery beyond genic regions to for example other *cis*-regulatory elements or unbiased genome-wide scans will potentially provide opportunities to identify the optimal clinically useful 5hmC biomarkers and enhance our knowledge of their biological relevance. Another challenge is that circulating cfDNA can originate from various sources; therefore, the genome-wide analysis of cfDNA is possibly hampered by the effect of genetic or epigenetic heterogeneity. A comprehensive cross-tissue comparison will be needed to establish highly tissue-specific 5hmC features in cfDNA [87]. Well-controlled animal models such as the patient-derived xenograft mouse model can also be utilized to evaluate experimentally the tumor relevance of 5hmC signals in cfDNA [45, 88]. Moreover, emerging technologies such as single-cell epigenetic assays could help determine the contributions of cfDNA from various sources [89]. Furthermore, considering their distinct biological functions and genomic distributions, integrating both 5mC and 5hmC modification markers together with nucleosome foot printing in the future would be promising to maximize the detection sensitivity in early-stage cancer and tissue-of-origin [90]. Finally, since the current 5hmC cancer biomarker discovery studies are generally small in sample size, future better statistically powered case–control and longitudinal studies will be necessary to identify more reliable 5hmC biomarkers, and evaluate the relationships between 5hmC and various potential confounding factors as well as the dynamic changes of 5hmC in patients (e.g., after treatment), thus facilitating the clinical applications of this promising tool in cancer precision medicine.

## Abbreviations

ctDNA: circulating tumor DNA
cfDNA: cell-free DNA
5mC: 5-methylcytosine
5hmC: 5-hydroxymethlcytosine
5fC: 5-formylcytosine
5caC: 5-carboxylcytosine
CTC: circulating tumor cell
AFP: alpha-fetoprotein
TNM staging: tumor staging based on primary tumor (T), reginal lymph nodes (N) and distant metastasis (M)
Tet: ten-eleven translocation
DMR: differentially methylated region
TAB-Seq: tet-assisted bisulfite sequencing
oxBS-Seq: oxidative bisulfite sequencing
RRBS: reduced representation bisulfite sequencing
RRHP: reduced representation 5-hydroxymethylcytosine profiling
CRC: colorectal cancer
AUC: area under the receiver operating characteristic curve
GC: gastric cancer
CEA: carcinoembryonic antigen
CA19-9: cancer antigen 19-9
CA125: carbohydrate antigen 125
NSE: neuron-specific enolase
EC: esophageal cancer
LC: lung cancer
LDCT: low-dose computed tomography
NSCLC: non-small-cell lung cancer
MM: multiple myeloma
PE: paired-end
